# Molecular characterization of human *Cryptosporidium* spp. isolates after an unusual increase in late summer 2012

**DOI:** 10.1186/s13071-016-1397-5

**Published:** 2016-03-10

**Authors:** Jeroen H. Roelfsema, Hein Sprong, Simone M. Cacciò, Katsuhisa Takumi, Michiel Kroes, Wilfrid van Pelt, Laetitia M. Kortbeek, Joke W. B. van der Giessen

**Affiliations:** Centre for Infectious Disease Control Netherlands, National Institute for Public Health and the Environment (RIVM), Bilthoven, The Netherlands; Department of Infectious, Parasitic and Immunomediated Diseases Istituto Superiore di Sanità, Rome, Italy

**Keywords:** *Cryptosporidium*, Parasite, GP60, Genotyping, Gastroenteritis, Population genetics

## Abstract

**Background:**

During the late summer 2012, a number of medical microbiological laboratories (MMLs) reported an unusual increase in cases of cryptosporidiosis, a gastrointestinal infection caused by the protozoan parasites *Cryptosporidium* spp. Prompted by this signal, the National Institute of Public Health and the Environment (RIVM) started an epidemiological investigation into possible causes. Simultaneously, samples diagnosed at MMLs were sent to RIVM for genotyping, aiming to further identify the possible source of the increase.

**Methods:**

Genotyping was performed by sequencing a fragment of the GP60 gene. Additional genotyping was performed on a subset of samples using six microsatellite markers. Population genetic analysis was performed using BEAST.

**Results:**

The majority of the samples were typed as *C. hominis*, and a single GP60 genotype (IbA10G2) largely predominated. Genotyping microsatellite markers further supported the circulation of a single genetic type. Population genetic analysis with genotypes found in previous years is inconsistent with a decrease in effective population size.

**Conclusions:**

The conclusion of this finding is that the rise reflects more an overall increase and not a common source outbreak.

## Background

*Cryptosporidium* spp. are protozoan parasites and are widespread in a diverse range of hosts such as mammals including humans, birds and fish. In the host *Cryptosporidium* spp. can cause gastro-enteritis with symptoms of diarrhoea, abdominal pain, nausea and fever [1]. Because of the global distribution of *Cryptosporidium* spp., these parasites can infect humans everywhere around the world.

There are more than twenty recognized species of *Cryptosporidium* [2, 3]. These species differ remarkably in their host specificity. Indeed, some species have a strong preference for a limited range of hosts, such as *C. hominis*, found almost exclusively in humans, whereas others, such as *C. ubiquitum* will infect a very broad range of hosts including rodents, cattle and humans [4, 5]. Humans are mostly infected by *C. parvum* and *C. hominis* [6–9]. *C. hominis* was first recognized as a distinct strain of *C. parvum*, only found in humans. Other *C. parvum* strains are not restricted to human hosts and can also be found in cattle and other animals [10, 11]. Later, *C. hominis* was recognized as a distinct species [4].

Human cryptosporidiosis in Europe occurs with seasonal peaks, particularly in August and September [12]. Seasonal peaks in The Netherlands have been reported: an increase of cryptosporidiosis in the spring, mainly caused by *C. parvum* and a peak late in the summer and the start of the autumn, caused both by *C. parvum* and *C. hominis* [7]. Detailed reports of seasonal peaks in the UK have been published [13–15]. Similar patterns have been described in other parts of the world as well, for instance in California (USA) and New Zealand [16, 17].

Next to sporadic cases, outbreaks of cryptosporidiosis can occur, often through contact with contaminated water containing infectious oocysts. Outbreaks of cryptosporidiosis as a result from a single source contamination occur occasionally in western European countries. Such outbreaks have recently been reported in Norway, Sweden and Spain [18–20]. Typical sources can be food, contaminated swimming pools or recreational water. Water-borne outbreaks are frequently reported worldwide [21, 22].

In 2012 a number of medical microbiological laboratories (MMLs) reported an unusually high increase of cases of cryptosporidiosis starting in August. A rise of cryptosporidiosis in August is to be expected but the diagnostic laboratories reported an increase beyond the ordinary. Based on collected data from eight laboratories that used the same detection method since 2010, a rise of more than three times of the number of patients compared to previous years was observed [23]. The National Institute of Public Health and the Environment (RIVM) also started a case–control study to identify possible sources of *Cryptosporidium* contamination but found no evidence of a single source outbreak [23]. Additionally, the MMLs sent in the *Cryptosporidium*-positive samples for genotyping, intended to assist the epidemiological investigation. Here, we present the results of a molecular characterization of the *Cryptosporidium* spp. isolates during that late summer increase.

## Methods

### Sample collection

Eighteen MMLs sent in *Cryptosporidium* spp.-positive samples, starting in August 2012. The majority were collected in August but some samples were already collected in July. Sample collection continued until the beginning of December. These samples were sent to the RIVM as either stool or DNA extracts. In case stool samples were received, we isolated DNA using the High Pure PCR template DNA isolation kit from Roche (Almere, The Netherlands) according to the manufacturer’s instructions.

### Species determination

We performed a real-time duplex PCR with dual labeled probes on a Roche LightCycler 480 apparatus to determine whether the species was *C. parvum* or *C. hominis*. We used a combination of a PCR on *C. parvum*, developed by Hadfield et al. [24] and a PCR we developed specifically for *C. hominis*. The *C. parvum* specific PCR targets a gene for a hypothetical protein and the *C. hominis* PCR targets part of the GP60 gene. The primers and probes are listed in Table 1. Each 25 μl reaction contained 10 picomoles (pmol) of each *C. parvum* primer, CRULib13F and CRULib13RCp and 7.5 pmol of probe CRULib13TMCp, 15 pmol of each *C. hominis* primer, ChomGP60F and ChomGP60R and 10 pmole probe ChomGP60Tp. We used the LightCycler Taqman master kit from Roche. Amplification took 45 cycles with 10 s denaturing at 95 °C, 20 s annealing at 60 °C and 20 s extension at 72 °C.

**Table 1:** List of primers and probes used in this study. Primers AL3531 and AL3535 are used in the first round and primers AL3532 and AL3534 are nested primers used in the second round to amplify part of the GP60 gene. The primers CRULib13F and CRULib13RCp in combination with probe CRULib13TMCp amplify and detect specifically a hypothetical gene of *C. parvum* whereas primers ChomGP60f, ChomGP60r and probe ChomGP60Tp amplify part of the GP60 and specifically detect *C. hominis*

Name	Sequence	5’label	3’label
ATGFOR	atgagattgtcgctcattatc		
AL3533REV	agatatatcttggtgcg		
AL3531	atagtctccgctgtattc		
AL3535	ggaaggaacgatgtatct		
AL3532	tccgctgtattctcagcc		
AL3534	gcagaggaaccagcatc		
CRULib13F	tccttgaaatgaatatttgtgactcg		
CRULib13RCp	ttaatgtggtagttgcggttgaac		
CRULib13TMCp	tatctcttcgtagcggcgta	Vic	MGB-NFQ
ChomGP60F	aaagaacaatgaagaaagccaaa		
ChomGP60R	ggtagaaggttgggtagcactct		
ChomGP60Tp	tcaaggtggctccaaaggagacg	Texas Red	BHQ2

### GP60 genotyping

Genotyping was performed by sequencing a fragment of the GP60 gene. We amplified a fragment of approximately 500 bp using primers ATGFOR and AL3533REV [25]. We also analysed 22 samples using a nested PCR with primers AL3531 and AL3535 for the first round and AL3532 and AL3534 for the second round leading to a fragment of approximately 850 bp [26]. Primers are listed in Table 1. PCRs were performed using Qiagen HotStarTaq (Qiagen, Venlo, The Netherlands). The products were analyzed on a 1.5 % agarose gel, visualized by GelRed^™^ Nucleic Acid Gel Stain from Biotium (Hayward, CA, USA). PCR products were treated with ExoSAP-IT from USB (Cleveland, USA) according to the manufacturer’s instructions. We sequenced the PCR products on a 3700 automated sequencer from Applied Biosystems (Nieuwerkerk a/d IJsel, The Netherlands), using the Big Dye Terminator kit according to the manufacturer’s instructions.

### Microsatellite analysis

To further investigate genetic variability of parasite isolates, six micro- and mini-satellite markers were analyzed. These markers correspond to the MS1, MS9, TP14, MM5, MM18, and MM19 loci. The MS1 marker contains a GGTGGTATGCCA repeat in the heat shock protein 70 gene located on chromosome 2. The MS9 marker contains a TGGACT repeat in a 2016 bp gene encoding a hypothetical protein located on chromosome 5. The TP14 marker contains a CAA repeat in a 8421 bp gene encoding a hypothetical protein located on chromosome 8. The MM5 marker contains a TCCTCCTCT repeat within a 11,418 bp gene located on chromosome 6. The MM18 marker contains a GGACCA repeat in a 5004 bp gene, also located on chromosome 8. The MM19 marker contains a GGAGCT repeat in a 7230 bp gene, again located on chromosome 8. PCR was performed as previously described [27]. The size of each PCR product was estimated by electrophoresis on a capillary apparatus (QiaXcel; Qiagen, Milan, Italy) by comparison to size standards. Each allele was assigned a unique number indicating the estimated size in base pairs.

### Estimating genetic diversity

The genetic diversity of a certain species can be used as an estimate for its effective population size [28]. Spatial or temporal changes in the population size of an infectious agent may reflect the dynamics of the prevalence of the disease (23382432, 23244453, takumi submitted 2015). DNA sequences of GP60 from two different time points were aligned using the multiple alignment software MAFFT [29]. Subsequently, the alignments were analyzed by a molecular evolution approach using the software Bayesian Evolutionary Analysis by Sampling Trees (BEAST) [28]. We set Hasegawa-Kishino-Yamamoto (HKY) model of DNA evolution and a constant mutation rate for each site of the GP60 sequences. Each simulation took 1,000,000 iterations. We discarded 10 % burn-in and used the rest of posterior samples for the constant population size as a quantification of the genetic diversity. A criterion for a satisfactory convergence for this parameter was an effective sample size greater than 200.

We tested whether the DNA sequences sampled in 2012 indicate a change in the estimated genetic diversity of *C. hominis* compared to the previous years. For this, we subtracted the posterior samples for the constant population sizes based on the sequences collected between 2003 and 2005 [7] from the posterior samples based on the sequences collected in 2012 for the current study. Proportion of positive differences is an estimate for the probability that the genetic diversity of *C. hominis* increased in 2012 compared to the previous years [30].

## Results

### Origin of samples

The unusually high increase of cryptosporidiosis was reported by MMLs in The Netherlands. Together, these laboratories cover most of The Netherlands. A total of 507 samples were received and analysed. Table 2 shows the origin of samples.

**Table 2:** Overview of samples sent to the RIVM. The samples came from different regions of The Netherlands. The middle and western part of The Netherlands is highly urbanised

Region	Samples	Typed	*C. hominis*	*C. parvum*	IbA10G2	Other
North West	71	46 (100 %)	44 (96 %)	2 (4 %)	36 (78 %)	10 (22 %)
West	29	15 (100 %)	14 (93 %)	1 (7 %)	14 (93 %)	1 (7 %)
Middle	138	65 (100 %)	59 (91 %)	6 (9 %)	53 (82 %)	12 (18 %)
South	89	55 (100 %)	51 (93 %)	4 (7 %)	47 (85 %)	8 (15 %)
South East	115	42 (100 %)	33 (79 %)	9 (21 %)	30 (71 %)	12 (29 %)
East	34	25 (100 %)	23 (92 %)	2 (8 %)	22 (88 %)	4 (16 %)
North East	31	21 (100 %)	19 (90 %)	2 (10 %)	16 (76 %)	5 (24 %)
Total	507	269 (100 %)	243 (90 %)	26 (10 %)	218 (81 %)	52 (19 %)

### Species identification by real-time PCR

Using a duplex real-time PCR, we determined whether the samples contained *C. hominis* or *C. parvum*. Out of the 507 samples tested, 410 (81 %) could be typed at the species level, of which 360 (88 %) were *C. hominis* and 50 (12 %) were *C. parvum*.

### Genotyping the samples using GP60 sequences and microsatellite markers

First, genotyping of the *Cryptosporidium* samples was performed using the GP60 gene. Approximately half of the 507 samples (*n* = 269) were typed. Genotypes are listed in Table 3. Nearly all samples, 90 % (*n* = 243), were *C. hominis*, which is concordant with the results from the real-time PCR assay. The predominant *C. hominis* genotype was IbA10G2, found in 81 % (*n* = 218). In total, 16 different genotypes were found, and most of these were represented by only one or two samples (Table 3). The two most common genotypes after IbA10G2 were the *C. hominis* type IaA15 (*n* = 21) and the *C. parvum* type IIaA15G2R1 (*n* = 11).

**Table 3:** GP60 genotypes found in isolates from the present study and from 2003 and 2005 [7]

Species	GP60 genotype	2012		2003–2005 (Wielinga *et al*.)	
		Number	Percentage	Number	Percentage
*C. hominis*	IbA10G2	218	81.04	65	82.28
*C. hominis*	IaA14R3	21	7.81		0
*C. hominis*	IeA11G3T3	3	1.12		0
*C. hominis*	IaA15	1	0.37		0
*C. hominis*	IaA24G1R1	1	0.37		0
*C. hominis*	IfA12G1	1	0.37		0
*C. hominis*	IcA5G3R2	0	0	1	1,27
*C. hominis*	IdA14	0	0	1	1,27
*C. hominis*	IdA17	0	0	1	1,27
*C. parvum*	IIaA15G2R1	11	4.09	8	10,13
*C. parvum*	IIaA17G2R1	3	1.12		0
*C. parvum*	IIaA17G1R1	2	0.74	1	1,27
*C. parvum*	IIcA5G3	2	0.74		0
*C. parvum*	IIdA17G1	2	0,74		0
*C. parvum*	IIaA16G3R1	1	0,37		0
*C. parvum*	IIdA18G1	1	0,37	1	1,27
*C. parvum*	IIdA19G1	1	0,37		0
*C. parvum*	IIdA22G1	1	0.37		0
*C. parvum*	IIdA15G1	0	0		0
*C. parvum*	IIdA16G1	0	0	1	1,27

The unusually high increase of cryptosporidiosis was reported by microbiological diagnostic laboratories that cover most of The Netherlands. When the geographical origin of the different genotypes was analysed, little difference between the various regions was found (Table 2). The increase of cases was observed throughout the country, and the IbA10G2 genotype predominated in all regions.

A panel of 20 randomly selected samples with the IbA10G2 genotype was further genotyped at six microsatellite markers [27]. Out of the 20 samples, 8 were typed at all markers, 8 were typed at most but not all markers, and 4 yielded no amplification. At each of the markers tested, only one allele was found, with estimated size of 374 bp (locus MS1), 261 bp (locus MS9), 261 bp (locus TP14), 210 bp (locus MM5), 233 bp (locus MM18) and 224 bp (locus MM19). Therefore, a single multi-locus genotype (MLG) characterized these 16 *C. hominis* isolates from 2012.

### Analysing genetic diversity

The genetic diversity of the isolates sharing the IbA10G2 genotype was analysed further with samples with the IbA10G2 genotype that were collected in previous years [7]. A total of 32 samples was analysed with the same set of microsatellite markers, except for marker MS9. Of these, 23 were fully genotyped, 5 were partially genotyped and 4 were negative. The estimated size of the alleles at each locus were identical to those found for the IbA10G2 samples collected in 2012.

The mean genetic diversity of the *C. hominis* isolates from 2012 was estimated to be 0.022 (95 % highest posterior density (HPD) = 0.015–0.029), whereas it was 0.015 (95 % HPD = 0.010–0.024) for the *C. hominis* isolates collected during the previous epidemiological investigation. The estimated probability that the genetic diversity of *C. hominis* increased in 2012 compared to the previous years was 0.88, thus the probability that it decreased or remained constant was 0.12.

## Discussion

In August 2012, an unusual rise of the number of cryptosporidiosis cases occurred in The Netherlands. At the early stage, it was unclear whether such increase was due to the known seasonality of this infection, i.e. to the usual summer peak, or to an ongoing, undetected outbreak. In the same period, an increase in cases was also noticed in Germany and in the UK. As in the Netherlands, the dominant species in the UK was *C. hominis*. The epidemiological study into the cause of the increase did not reveal a clear common source [23]. Additionally, it seemed unlikely that a single contamination source, such as a single type of food, could be responsible for the observed increase not only across The Netherlands, but also in Germany and the UK.

To gain additional insights, a genetic characterization of isolates from The Netherlands was undertaken. This revealed that a specific GP60 genotype was clearly predominating, although other GP60 genotypes were also found, adding up to 19 % of the total. The initial goal was to refine the case definition for the epidemiological survey in order to strengthen the data leading to a cause. Statistically significant risk factors, however, were not found [23]. Nevertheless, the finding that 81 % of all cases were caused by the IbA10G2 genotype may be considered as compatible with a major single contamination source. We, therefore, analysed some of the IbA10G2 samples with microsatellite markers. The analysis of six microsatellite markers revealed no length polymorphism among samples from 2012 or from previous years, suggesting that the IbA10G2 genotype from the Netherlands represents a genetically homogeneous strain.

Does the high percentage of the IbA10G2 genotype in Dutch patients simply reflect its overwhelming presence in the environment? We do not know, but we do know from previous data from The Netherlands and the neighbouring region of East Flanders, that this has been the most commonly identified *C. hominis* genotype in the last years. Wielinga et al. 7 presented GP60 genotype data on *Cryptosporidium* spp. found in clinical samples from The Netherlands between the years 2003 and 2005. The study by Wielinga et al. was conducted differently from this study. We aimed to type as many samples as possible from patients diagnosed at a large number of laboratories across the country during a limited period of approximately 5 months. On the other hand, Wielinga et al. collected samples from a limited number of MMLs, not covering the entire country, and during several years. Nevertheless, in these samples the IbA10G2 genotype was also the predominant genotype, found in 65 out of the 80 samples from 2003 to 2005. The 2009 study from Belgium also confirms the predominance of the IbA10G2 genotype, although this was less striking with 10 of 24 samples [31].

The mean genetic diversity of the 2012 samples was higher than that of the samples from 2003 to 2005. Although the estimated probability for the increase in the genetic diversity was less than the threshold of 0.95 by 0.07, the change took place at the same period in which the incidence rate also increased. This is in line with our previous findings that indicated that the genetic diversity of GP60 may be an independent and complementary measure for quantifying disease incidence [32].

In an outbreak situation, where there is only a limited number of genotypes involved in the infection of a large population, one might have expected a short-term *decrease* in genetic diversity rather than an increase. Detailed genetic analyses of (future) outbreaks of *Cryptosporidium* spp. may help us to be able to distinguish between upsurges and unapparent outbreaks of cryptosporidiosis.

## Conclusions

In conclusion, the increase in the number of *Cryptosporidium*-positive patients in 2012 was predominantly caused by *C. hominis* genotype IbA10G2. The similar distribution of *Cryptosporidium* genotypes was also identified in previous years. Both the mean genetic diversity of *C. hominis* and the number of patients increased compared to the previous years. The conclusion of this finding is that the rise reflects more an overall increase and not a common source outbreak.
